# The Effect of Pyriproxyfen as a “Population Growth Regulator” against *Aedes albopictus* under Semi-Field Conditions

**DOI:** 10.1371/journal.pone.0067045

**Published:** 2013-07-02

**Authors:** Shin-ya Ohba, Kazunori Ohashi, Endang Pujiyati, Yukiko Higa, Hitoshi Kawada, Nobuaki Mito, Masahiro Takagi

**Affiliations:** 1 Biological Laboratory, Faculty of Education, Nagasaki University, Nagasaki, Japan; 2 Health and Crop Sciences Research Laboratory, Sumitomo Chemical Co. Ltd., Hyogo, Japan; 3 Department of Vector Ecology and Environment, Institute of Tropical Medicine, Nagasaki University, Nagasaki, Japan; Centro de Pesquisas René Rachou, Brazil

## Abstract

An insect growth regulator, pyriproxyfen, has been used for the control of a range of pest insects, including mosquitoes. Pyriproxyfen is effective in inhibiting adult emergence and sterilizing adult females. The Asian tiger mosquito, *Aedes albopictus* (Skuse), is an important vector of dengue and chikungunya, and is expanding its distribution throughout Europe and the Americas. In the present study, we evaluated the impact of pyriproxyfen-treated bed nets on population growth of *Ae. albopictus* under semi-field conditions, using 6 small microcosms. We created microcosms containing breeding sites to simulate the natural ecosystem of vector mosquito and installing miniature bed net treated with 350 mg/m^2^ pyriproxyfen in Experiment I and 35 mg/m^2^ in Experiment II. For each experiment, we also established microcosms installing untreated polyethylene net (untreated control). The installing nets were provided with artificially torn holes, to simulate damage and allow mosquitoes to penetrate. We released 100 pairs of *Ae. albopictus* into each microcosm, and allowed them to feed on a mouse under the bed nets at approximately 1-week intervals. In comparison with the untreated control microcosms, the number of eggs laid by the released adults in the pyriproxyfen-treated microcosms was significantly lower in both Experiment I and II. Moreover, egg hatchability was significantly suppressed and pupal mortality was increased. Our results indicate that tarsal contact with pyriproxyfen has been shown to suppress egg production and hatchability in adult females and the auto-dissemination of pyriproxyfen into larval breeding sites by adult mosquitoes, through contact with pyriproxyfen-treated polyethylene bed nets, may suppress the mosquito population density.

## Introduction

The Asian tiger mosquito, *Aedes albopictus* (Skuse), is an epidemiologically important vector for the transmission of many viral pathogens (e.g., dengue fever, chikungunya fever, West Nile virus, yellow fever virus, and St. Louis encephalitis [1], [2]), and also several filarial nematodes (e.g., *Dirofilaria immitis* [3]). *Aedes aegypti* is recognized as the primary vector for dengue and dengue hemorrhagic fevers, while *Ae. albopictus* is known to be a secondary vector [2]. The shipment of used tires is a major cause of the global expansion of *Ae. albopictus* [2], [4], which can outcompete and even eradicate other species with similar breeding habitats, following its dispersal to new regions and biotopes [5]. Intrinsic (e.g., high physiological plasticity and strong competitive aptitude) and extrinsic factors (e.g., globalization) have made *Ae. albopictus* an important vector of dengue and chikungunya fever [6], [7]. Thus, the control of mosquito vectors must consider not only *Ae. aegypti*, but also *Ae. albopictus*.

To date, reduction in the population density of vector mosquitoes has been the only treatment option for controlling the transmission of dengue virus in the human population. There is no promising prophylactic vaccine [8]. *Aedes albopictus* feed on human blood, and immature larvae have been found in several different natural and human-made containers (e.g., bamboo stump-pools, artificial containers, used tires [9]–[11]). Control measures for the closely related species, *Ae. aegypti*, include the use of larvivorous fish (e.g., *Poecilia reticulate*) and copepods (*Mesocyclops*), chemical larvicides, and adoption of preventive practice by local residents (e.g., use of covers on water-storage containers, frequent exchange of stored water, or elimination of unnecessary containers) [12]–[14]. These measures target the mosquito larvae or pupae, in an attempt to reduce vector density.

Pyrethroids which are organic compounds similar to natural pyrethrins produced by the flowers of pyrethrum (*Chrysanthemum cinerariaefolium* and *C. coccineum*), is the general term for a group of synthetic chemicals. The high knockdown and killing activity of pyrethroids means that they are used as the predominant insecticides for vector control. Globally, pyrethroids comprise 40% of insecticides used annually for indoor residual spraying malaria vectors, and 100% of the World Health Organization (WHO) recommended insecticides for the treatment of bed nets [15]. Pyrethroid-treated bed nets (long-lasting insecticidal nets, LLINs) have proven effective against malaria vectors, and are widely used in malaria-endemic countries. The use of LLINs for bed nets and house screening has been reported to reduce *Aedes* populations and dengue fever transmission [16]–[19]. However, pyrethroid resistance remains a major problem in vector control. Pyrethroid resistance of *Ae. albopictus* and *Ae. aegypti* has been well documented [20]–[25]. Thus, alternative chemicals are required for improved control of dengue and malaria.

The insect growth regulator (IGR), pyriproxyfen, is a juvenile hormone analog with low toxicity to mammals. Pyriproxyfen inhibits metamorphosis and embryogenesis in several insects [26]. When delivered into mosquito larval breeding sites at low concentrations, it is highly effective in inhibiting adult emergence [27]–[30]. However, the use of pyriproxyfen against dengue vectors is hindered by the large number of cryptic larval habitats. Thus, auto-dissemination of the chemical into larval breeding sites by adult mosquitoes has been considered [14], [31], [32]. Itoh et al. [33] suggested utilization of blood-fed *Ae. aegypti* females as a vehicle for the horizontal transfer of pyriproxyfen from adult resting sites into larval habitats. This unique method was established against container-breeding mosquitoes, under laboratory and field conditions [14], [31]–[35]. Field tests in an Amazonian city (Iquitos, Peru; [14]) yielded promising results. The effectiveness of using *Ae. albopictus* adult females as a vehicle for the transfer of pyriproxyfen to larval habitats has been evaluated under field conditions in Rome [36].

Itoh et al. [33] and Ohashi et al. [37] demonstrated a significant reduction in fecundity of mosquito females, following tarsal contact with a pyriproxyfen-treated surface. Thus, two possible pyriproxyfen-mediated reproductive suppression effects may exist [33]: (1) blood-fed females exposed to pyriproxyfen may carry the chemical to the larval habitats, thereby inhibiting adult emergence; and (2) tarsal contact with pyriproxyfen may inhibit egg maturation in adult mosquitoes. These effects represent potential measures for combating pyrethroid resistant dengue vectors. We propose, therefore, that bed nets treated with an insect growth regulator may not only offer personal protection from mosquito biting, but may also provide a reproductive suppression effect against vector mosquitoes.

The objectives of the present study were to evaluate the effects of pyriproxyfen-treated polyethylene bed nets on populations of a pyrethroid-susceptible strain of *Ae. albopictus*, under semi-field conditions. Here, we compare our results with those of previous studies, and discuss the advantage of our technique for controlling container-breeding mosquitoes.

## Materials and Methods

### Preparation of Pyriproxyfen-treated Netting

We used non-insecticidal net composed of 195-denier monofilament polyethylene, with a mesh size of 75 holes/square inch, equivalent to Olyset® Net (Sumitomo Chemical Co. Ltd., Tokyo, Japan). Technical-grade pyriproxyfen, registered as Sumilarv® (Sumitomo Chemical Co. Ltd.), was diluted with isopropyl alcohol at concentrations of 1.0% and 0.1% (w/v) for Experiments I and II, respectively. Square netting (length 50 cm×width 50 cm×height 50 cm), simulating a miniature bed net, was dipped in each concentration of the pyriproxyfen solution for 1 h and dried overnight. Chemical analysis using gas chromatography (GC-2010; Shimazu Co., Kyoto, Japan) revealed that net treated with 1.0% and 0.1% pyriproxyfen retained approximately 350 mg/m^2^ and 35 mg/m^2^ of the chemical, respectively.

### Microcosms

**Figure 1:**
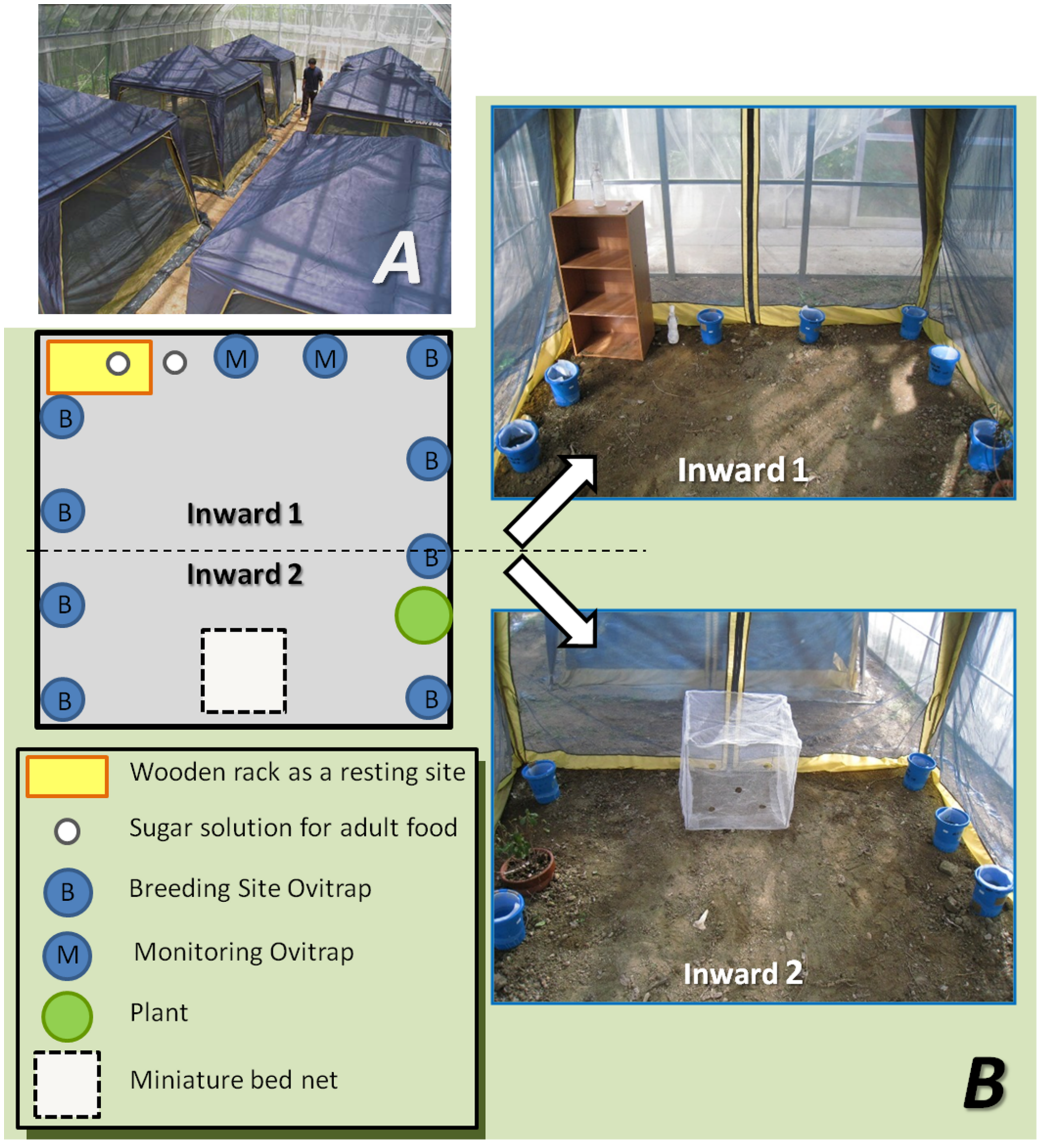
Diagram and photographs of the semi-field experiment. (A) Overall view of the greenhouse. (B) Diagram of the microcosm. *Breeding Site Ovitraps*: 8 ovitraps were assigned as larval breeding sites and for counting the number of eggs and pupae; *Monitoring Ovitraps*: 2 ovitraps were used to investigate the egg-hatching rate and pupal mortality. The egg-hatching rate was monitored on filter paper under laboratory conditions. Pupal mortality arising through horizontal transfer of pyriproxyfen by adult females was evaluated in a bioassay under laboratory conditions, using water from the Monitoring Ovitraps.

We installed 6 small microcosms (length, 270 cm; width, 270 cm; height 227 cm) in a large greenhouse (16 m×10 m) at Nomozaki Experimental Station, Nagasaki University (32°35′N, 123°45′E; Figure 1-A). To investigate the effect of pyriproxyfen-treated bed nets on *Ae. albopictus* populations, we created microcosms installing pyriproxyfen-treated polyethylene net (pyriproxyfen treatment, *n* = 3), and microcosms installing untreated polyethylene net (control, *n* = 3).

We conducted 2 different microcosm trials against *Ae. albopictus*. Experiment I and II were carried out using nets treated with 1.0% and 0.1% pyriproxyfen described above, respectively. The average temperatures during Experiments I and II were 25.9°C (min, 24.0°C; max, 28.3°C) and 20.2°C (min, 12.2°C; max, 24.7°C), respectively.

**Figure 2:**
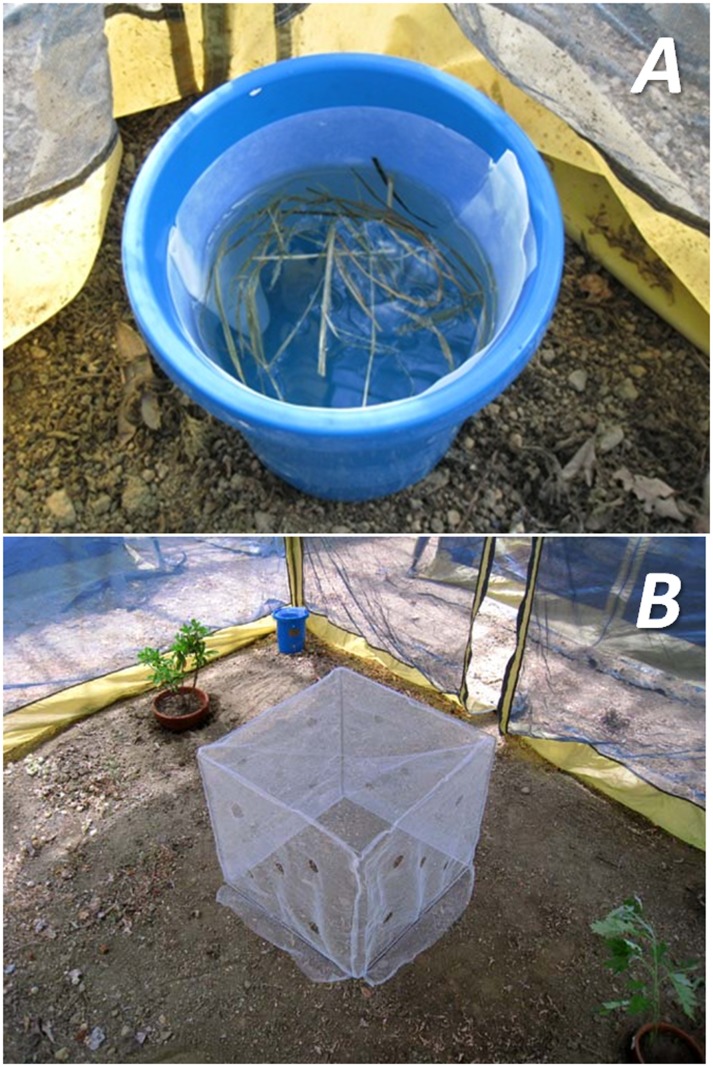
Ovitrap and polyethylene net installed in the microcosms. (A) Ovitrap lined with filter paper. Small amounts of hay (0.2 g) and larval food (3 mg) were placed in the water. (B) Polyethylene net (50 cm×50 cm×50 cm, with a mesh size of 75 holes/square inch, equivalent to Olyset® Net [Sumitomo Chemical Co. Ltd., Tokyo, Japan]) was placed in the microcosms. Note the 5 artificially torn holes (diameter 5 cm) on each panel. A restrained mouse was placed under the net, and the number of females landing for blood feeding was counted.

We used artificial (wooden rack) and natural (*Rhododendron pulchrum*) structures, to simulate the garden of a human house (Figure 1-B). Two 500-mL bottles, containing filter paper and 3% sugar solution, were placed beside the wooden rack to provide adult food. Ten ovitraps (height 18 cm, diameter 14.5 cm), containing 1.2 L of water, were placed in each microcosm. Ovitrap water contained 3 mg of a 1∶1 mixture of mouse pellet powder and dry yeast as standard larval food, and 0.2 g of hay to attract gravid females (Figure 2-A). A filter paper (width 5 cm) was aligned at water-surface level for ovipositional substance. Of the 10 traps, 8 (hereafter referred to as “Breeding Site Ovitraps”) were assigned as larval breeding sites, and for counting the number of eggs and pupae. The remaining 2 traps (hereafter referred to as “Monitoring Ovitraps”) were used to investigate the egg-hatching rate and pupal mortality described below. A miniature bed net (50 cm×50 cm×50 cm), with 5 artificially torn holes (diameter 5 cm) on each panel to simulate damage and allow mosquitoes to penetrate before and/or after blood feeding, was placed in each microcosm (Figure 2-B).

This experiment involves some minor stress or pain (short-duration pain) to mouse. No particular pain relief is necessary because it involves only minor pain, such as brief retention, or restraint.

### Release and Monitoring of Mosquitoes in the Microcosms

At the start of each trial, we released 100 pairs of laboratory-reared *Ae. albopictus* into each microcosm. The strain used (strain name: NG2002) was collected in Nagasaki in 2002, and maintained in the laboratory. Within the microcosms, mosquitoes were allowed to feed on a mouse at 0, 4, 11, and 20 d after release for Experiment I, and at 0, 6, 13, 21, 27, 34, and 44 d after release for Experiment II. Mosquitoes were in contact with the pyriproxyfen-treated netting before and/or after blood feeding. We monitored the *Ae. albopictus* populations on each day of blood feeding.

### Number of Adult Females

To estimate the adult population size, we counted the number of adult females landing on a mouse for blood feeding. The mouse (body length, 11 cm; weight, ca. 55 g) was restrained and placed under the net. Females entered the net through the artificially torn holes in the side panels and fed on the mouse for 2 h. The number of females landing on the mouse during the first hour were counted at 5-min intervals, and the population size was calculated using the area under the frequency trend curve according to Kiritani, Nakasuji and Manly’s method [38], [39]. With this method, *A_i_* is estimated by the trapezoidal rule according to the following formula
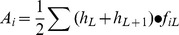
where *f_iL_* = the number of the female mosquitoes on a mouse from the samples taken on the *L*th occasion, which is at the end of the sampling intervals *h_L_*, there are *h_L_*_+1_ sampling intervals [39]. The number of adult females is then estimated by dividing *A_i_* by 9.07 (min), which represents the average blood-sucking time of the *Ae. albopictus* adult females used in the study. Almost all females finished their blood meal during the first hour, and therefore counts were not conducted during the second hour.

### Numbers of Eggs and Pupae

We counted the number of eggs laid on the filter paper in the 8 Breeding Site Ovitraps of each microcosm. After counting, the filter papers were submerged in the water of the Breeding Site Ovitrap, to allow the eggs to hatch. After submerging the paper, the Breeding Site Ovitrap was refilled by tap water to the initial level (1.2 L) if the water level had gone down due to evaporation. A new filter paper was placed inside the Breeding Site Ovitrap for the next observation. The number of pupae in each of the 8 Breeding Site Ovitraps was counted as an index of immature abundance. The mean numbers of eggs and pupae in the 8 Breeding Site Ovitraps were used to represent the average numbers of eggs and pupae for each microcosm.

### Egg-hatching Rate

We recorded the egg-hatching rate on the filter papers in the 2 Monitoring Ovitraps, under laboratory conditions. The papers were dried for 4 d and then submerged in water. The number of eggs hatching was counted daily for 14 d after submergence. The mean egg-hatching rate for the 2 Monitoring Ovitraps was used to represent the egg-hatching rate for each microcosm.

### Pupal Mortality

To evaluate potential contamination of ovitrap water through horizontal transfer of pyriproxyfen by females, we performed a bioassay using fourth instar, laboratory-reared *Ae. albopictus* larvae. We sampled 300 mL of water from each of the 2 Monitoring Ovitraps in the both pyriproxyfen-treated and control microcosms. We prepared three replications for each Monitoring Ovitrap. We placed 100 mL of the sampled water into a 200-mL plastic cup (diameter, 100 mm; height, 45 mm), and released 10 fourth instar laboratory-reared larvae into the water. The plastic cups were maintained at a temperature of 25°C temperature, under a 16-h light, 8-h dark light cycle. After 7 d, we recorded the adult emergence rate. In Experiment I, no larval food was supplied, while in Experiment II, 5 mg of standard larval food was placed in each of the cups. Pupal mortality was recorded with 3 replicates for each Monitoring Ovitrap, and the mean mortality for the 2 Monitoring Ovitraps (*n = *6) was used to represent the pupal mortality for each microcosm. The waters in the Monitoring Ovitraps in the Experiment I and II were collected and brought to laboratory at the end of experiment to determine pyriproxyfen concentrations. The pyriproxyfen in the water was extracted with hexane and analyzed for concentrations by a LC-MS/MS system (ACQUITY UPLC; Waters Corp., Milford, MA, USA, and 3200 Q TRAP; Applied Biosystems, Foster, CA, USA). In addition, the pyriproxyfen concentrations in the water of Monitoring Ovitraps were estimated from pupal mortality in the Experiment II by using the probit regression formula reported by Satho [40]. The pyriproxyfen concentrations in the Experiment I were not estimated because the pupal mortality in Control was high due to the food shortage for larvae.

### Statistical Analysis

The numbers of females on a mouse, eggs, pupae, pupal mortality, and egg-hatching rate of *Ae. albopictus* for the 2 treatments were compared using a repeated measures analysis of variance (ANOVA), with treatment (pyriproxyfen treatment and untreated control) as the between-subject factor, and time (each monitoring day) as the within-subject factor. If Mauchly’s test indicated a significant violation of the assumption of sphericity (*P*<0.05), significance levels for within-subject effects were calculated using Greenhouse–Geisser adjustment for the degrees of freedom (G-G correction) [41]. When a significant interaction was encountered for between-subject and within-subject effects or as necessary, separate one-way ANOVA tests were applied to the differences between treatments on each monitoring day. Log_10_ (*n* +1) transformations for the numbers of females on a mouse, eggs and pupae and arcsine square-root transformations for egg-hatching rate and pupal mortality were made to satisfy the assumptions of the ANOVA model. All statistical tests were conducted using AVOVA-KUN Version 4.3.2 [42] at R version 2.14.0 [43].

### Ethics Statement

This animal experimental protocol was reviewed and formally approved by the Animal Ethics Committee of the Nagasaki University (approval No. 1206250996).

## Results

### Effect of Pyriproxyfen-treated Netting on the Number of Adult Females

**Figure 3:**
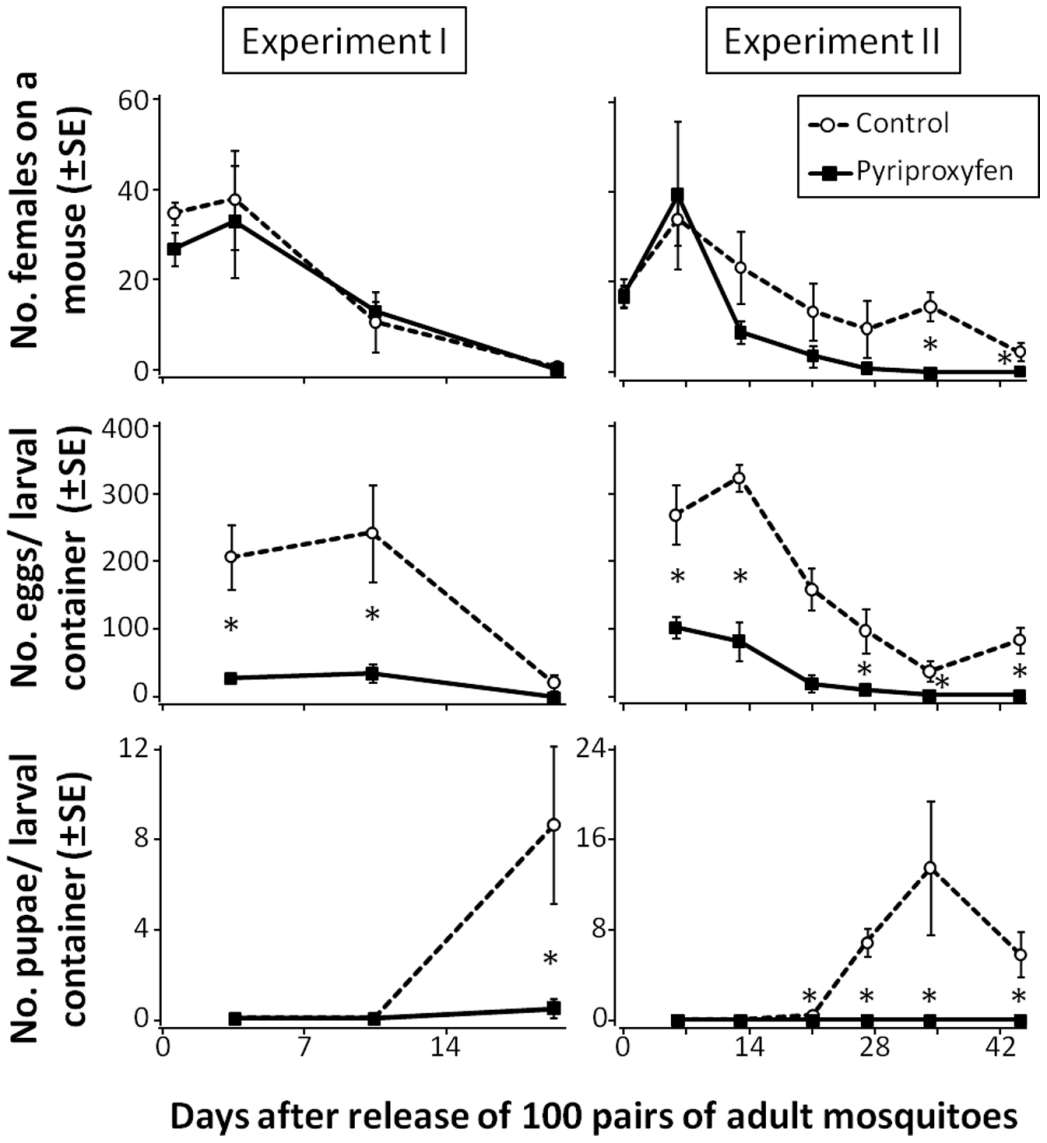
Abundance of *Aedes albopictus* in the control and pyriproxyfen treatment. Experiment I, 1% pyriproxyfen; Experiment II, 0.1% pyriproxyfen. *P<0.05, as analyzed by one-way ANOVA.

Repeated measures ANOVA for the number of adult females on a mouse revealed that time was significant in Experiment I, but that treatment (pyriproxyfen treatment and untreated control) and treatment-by-time interactions were not significant (treatment, *F*_1,4_ = 0.0125, *P* = 0.92; time, *F*_3,12_ = 40.06, *P* = 0.001; treatment×time, *F*_3,12_ = 0.17, *P* = 0.91). No effect of pyriproxyfen on the number of adult females was detected during the experimental period (Figure 3). In Experiment I, the number of adult females declined to almost zero for the pyriproxyfen treatment and untreated control (pyriproxyfen treatment, mean ± SE = 0.50±0.50; untreated control = 0.18±0.18; Figure 3). Experiment I was ended after 20 d. However, second-generation adults may have occurred in untreated control after 20 d, because of the increasing number of pupae at day 20 (Figure 3).

In Experiment II, all effects were significant (treatment, *F*_1,4_ = 38.13, *P* = 0.009; time, *F*_6,24_ = 14.29, *P*<0.001; treatment×time, *F*_6,24_ = 4.32, *P* = 0.0072). The number of adult females in Experiment II declined gradually over time, and then increased in the control at day 34 and day 44 (day 34, *F*_1,4_ = 189.54, *P* = 0.0001, day 44, *F*_1,4_ = 12.69, *P* = 0.024, one-way ANOVA,) after release. This increase was attributed to the emergence of second-generation adults from the Breeding Site Ovitraps.

### Effect of Pyriproxyfen-treated Netting on the Number of Eggs

Repeated measures ANOVA for the number of eggs in Experiment I revealed that treatment and time were significant, but that the treatment-by-time interaction was not significant (treatment, *F*_1,4_ = 22.75, *P* = 0.018; time, *F*_2,8_ = 14.39, *P* = 0.005; treatment×time, *F*_2,8_ = 0.001, *P* = 0.999; Figure 3). However, the number of eggs at day 20 did not differ between treatments (*F*_1,4_ = 4.17, *P* = 0.11, one-way ANOVA), because of the reduction in the number of mature adult females (Figure 3).

Repeated measures ANOVA for the number of eggs in Experiment II revealed that treatment, time, and the treatment-by-time interaction were significant (treatment, *F*_1,4_ = 39.33, *P* = 0.008; time, *F*_5,20_ = 13.62, *P*<0.001; treatment×time, *F*_5,20_ = 3.218, *P* = 0.004). Separate one-way ANOVA revealed that the number of eggs differed significantly between the pyriproxyfen treatment and untreated control at day 6, day 13, day 27, day 34 and day 44 (day 6, *F*_1,4_ = 17.69, *P* = 0.014; day 13, *F*_1,4_ = 15.79, *P* = 0.017, day 27; *F*_1,4_ = 9.14, *P* = 0.039; day 34, *F*_1,4_ = 20.26, *P* = 0.011; day 44, *F*_1,4_ = 281.34, *P*<0.001), and did marginally at day 21 (*F*_1,4_ = 7.08, *P* = 0.056).

### Effect of Pyriproxyfen-treated Net on the Number of Pupae

Repeated measures ANOVA for Experiment I revealed that treatment, time, the treatment-by-time interaction were significant (treatment, *F*_1,4_ = 18.01, *P* = 0.024; time, *F*_1, 4_ = 30.42, *P* = 0.012 (after G-G correction); treatment×time, *F*_1,4_ = 18.01, *P* = 0.024 (after G-G correction), G-G *ε* = 0.500; Figure 3). The number of pupae increased at day 20 after release. Separate one-way ANOVA showed that the number of pupae was lower in the pyriproxyfen treatment than in the untreated control at day 20 (*F*_1,4_ = 18.38, *P* = 0.013).

For Experiment II, all effects were significant (treatment, *F*_1,4_ = 96.12, *P* = 0.002; time, *F*_5,20_ = 19.07, *P*<0.0001; treatment×time, *F*_5,20_ = 18.16, *P*<0.0001). The number of pupae was significantly lower in the pyriproxyfen treatment than in untreated control. Separate one-way ANOVA revealed that the number of eggs differed significantly between the pyriproxyfen treatment and untreated control at day 21, day 27, day 34 and day 44 (day 21, *F*_1,4_ = 170.37, *P*<0.001; day 27, *F*_1,4_ = 37.03, *P* = 0.004; day 34, *F*_1,4_ = 24.76, *P* = 0.008; day 44, *F*_1,4_ = 15.79, *P* = 0.016).

### Effect of Pyriproxyfen-treated Netting on the Egg-hatching Rate

**Figure 4:**
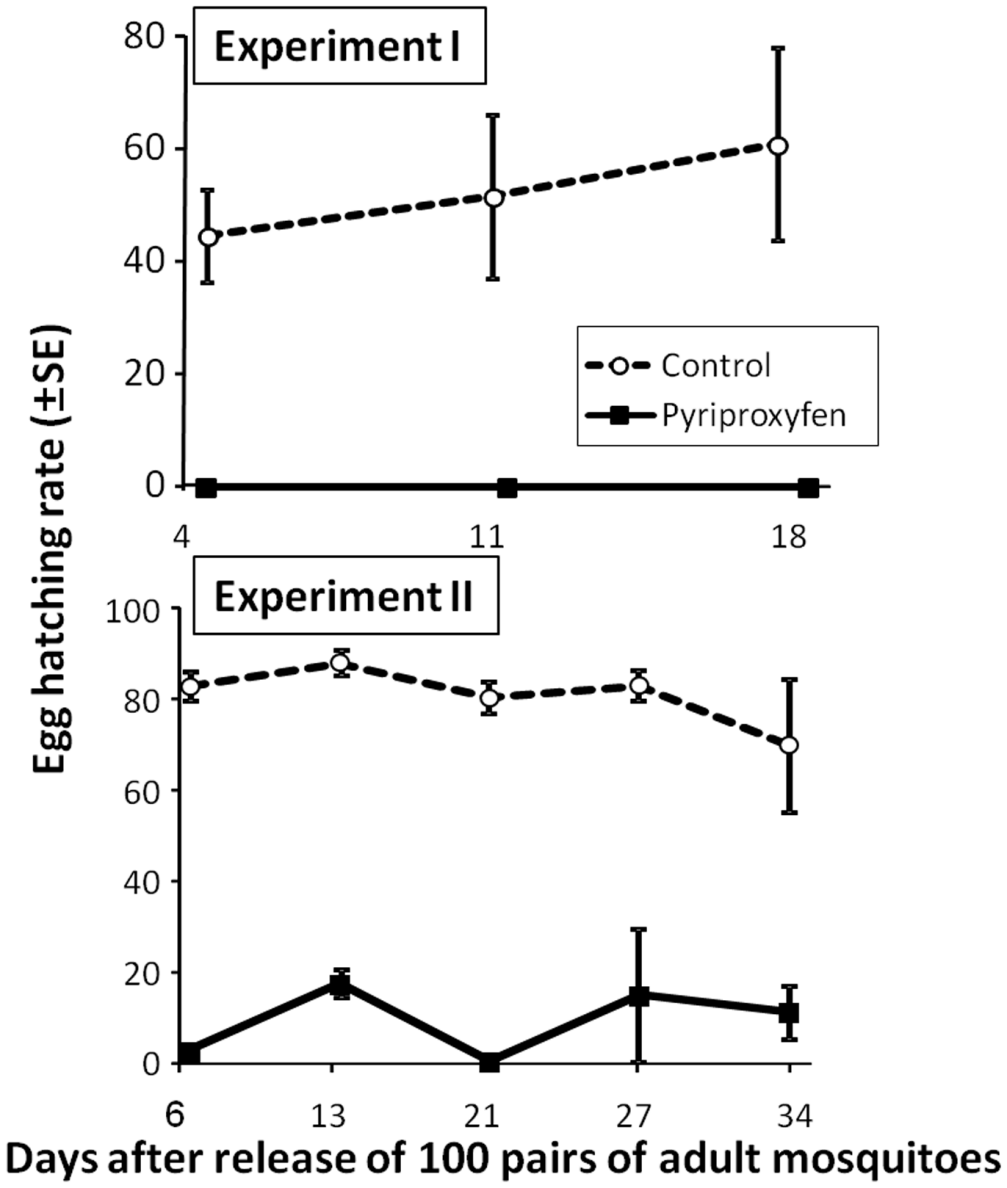
Egg-hatching rate of *Ae. albopictus* in the control and pyriproxyfen treatment. Repeated measures ANOVA revealed a significant difference (*P*<0.05) between the untreated control and pyriproxyfen treatment in Experiment I, and also in Experiment II. Data at day 44 in Experiment II was not shown here.

For Experiment I, none of the eggs collected from Monitoring Ovitraps in the pyriproxyfen treatment hatched (Figure 4). Repeated measures ANOVA revealed that treatment was significant, but that time and the treatment-by-time interaction was not significant (treatment, *F*_1,4_ = 3657.6, *P*<0.001; time, *F*_1,4_ = 1.65, *P* = 0.29 (after G-G correction); treatment×time, *F*_1,4_ = 0.29, *P* = 0.28 (after G-G correction), G–G *ε = *0.500). Meanwhile, for Experiment II, Repeated measures ANOVA revealed that treatment and time were significant, but that the treatment-by-time interaction was not significant (treatment, *F*_1,4_ = 630.26, *P*<0.001; time, *F*_2,8_ = 18.36, *P* = 0.003; treatment×time, *F*_2,8_ = 6.71, *P* = 0.30). The egg-hatching rate was significantly lower in the pyriproxyfen treatment than in the untreated control (Figure 4).

### Effect of Pyriproxyfen-treated Netting on Pupal Mortality

**Figure 5:**
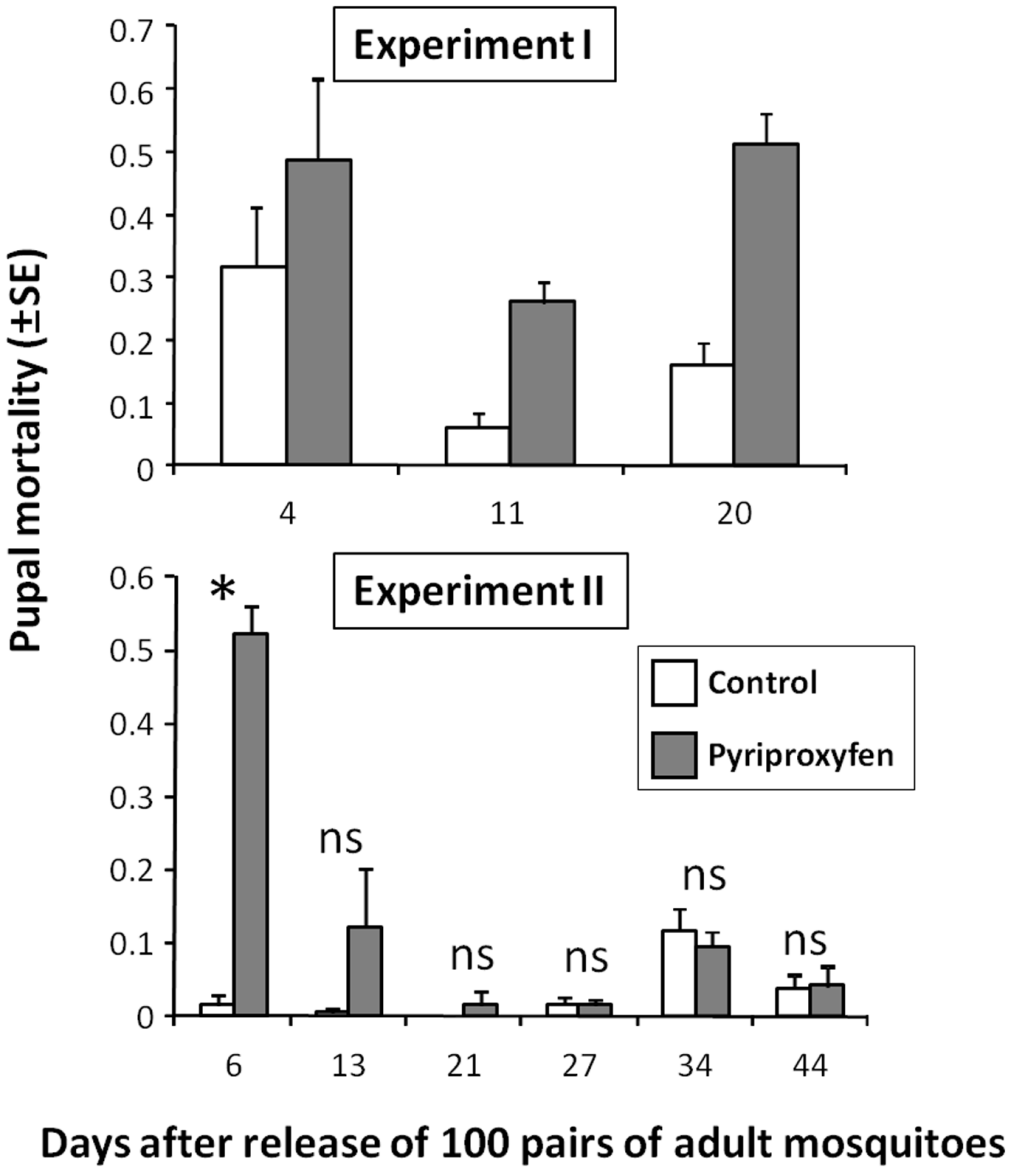
Pupal mortality of *Ae. albopictus* in the control and pyriproxyfen treatment. Repeated measures ANOVA revealed a significant difference between the untreated control and pyriproxyfen treatment in Experiment I, and also in Experiment II. *P<0.05 as analyzed by one-way ANOVA.

Repeated measures ANOVA revealed that treatment was significant, but that time and the treatment-by-time interaction were not significant (treatment, *F*_1,4_ = 14.43, *P* = 0.032; time, *F*_2,8_ = 4.64, *P* = 0.060; treatment×time, *F*_2,8_ = 0.437, *P* = 0.67). Throughout Experiment I, the pupal mortality measured in the water from the Monitoring Ovitraps was significantly higher in the pyriproxyfen treatment than in the untreated control (Figure 5). Larval food was not supplied in Experiment I, and therefore the higher mortality (even in the control) was probably the result of food shortage.

By contrast, for Experiment II, repeated measures ANOVA revealed that treatment, time, and the treatment×time interaction were all significant (treatment: *F*_1,4_ = 12.21, *P* = 0.040; time: *F*_5,20_ = 8.34, *P*<0.001; treatment×time: *F*_5, 20_ = 7.20, *P* = 0.001). Separate one-way ANOVA showed that the pupal mortality differed significantly between the pyriproxyfen treatment and the untreated control only on the first day of observation (day 6, *F*_1,4_ = 120.17, *P*<0.0001). Based on the pupal mortality, the pyriproxyfen concentration in the Monitoring Ovitraps at the first day of observation (day 6) was estimated at 0.082 ppb in the pyriproxyfen treatment. The amount was less than 0.001 ppb at day 13 and thereafter. Out of six water samples from Monitoring Ovitraps collected at the end of experiment I, two samples were detected pyriproxyfen at 0.07 ppb and 0.02 ppb by the chemical analysis using LC-MS/MS system, while another 4 samples were not detected pyriproxyfen at the detection limit of the system. At the end of Experiment II, all collected water samples were not detected pyriproxyfen.

## Discussion

The main objective of the present study was to determine the effect of pyriproxyfen-treated bed nets on *Ae. albopictus* populations, under semi-field conditions. We observed that the number of eggs and pupae generated from first-generation adults released into the microcosms of Experiment I and II were lower in the pyriproxyfen treatment than in the untreated control (Figure 3). However, there was no significant effect of pyriproxyfen on the number of adult females in either the pyriproxyfen treatment or the untreated control during the experimental period in Experiment I and the occurrence period of first generation (up to day 27 after release) in Experiment II (Figure 3). This slow-acting effect is a feature of pyriproxyfen, and allows adult mosquitoes to act as a vehicle for the transfer of pyriproxyfen to larval habitats. Our results indicate that pyriproxyfen can significantly reduce the numbers of *Ae. albopictus* eggs, pupae, larvae, and next-generation adults by: (1) inhibiting the egg production of adult females, following contact with pyriproxyfen-treated netting; and/or (2) causing the death of immature larvae, following transfer by adult females.

Tarsal contact with pyriproxyfen has been shown to suppress egg maturation in *Ae. aegypti* adult females, before or up to 24 h after blood feeding [33], [44]. Juvenile hormone is also known to regulate insect ovarian development. Shapiro et al. [45] demonstrated that a decline in juvenile hormone levels during or up to 36 h after blood feeding is necessary for normal egg development of *Ae. aegypti*. Topical application of juvenile hormone analogues onto the mosquito abdomen has been reported to reduce fecundity and egg fertility in *Ae. aegypti* [46]. In the present study, we observed that *Ae. albopictus* adult females penetrated the polyethylene bed net through artificially torn 5-cm holes (Figure 2-B), in order to feed on the blood of mouse. After feeding, the females rested on the inside of the netting. Thus, contact with pyriproxyfen-treated netting causes a disturbance in the hormonal regulation of ovarian development, resulting in reduced fecundity and egg hatchability. Previous laboratory studies revealed that contact with pyriproxyfen reduced the fecundity and egg hatchability of *Ae. aegypti* and *Ae. albopictus* (Ohashi et al. unpublished data). In the present study, we observed a significant decrease in fecundity and pupal density in the Breeding Site Ovitraps and egg hatchability in the Monitoring Ovitraps of Experiments I and II, following treatment with pyriproxyfen.

Previous studies have reported that pyriproxyfen adheres to the body of mosquitoes, and can be detected by chemical analysis [33], [47] and scanning electron microscopy [14]. In the present study, we did not confirm attachment of pyriproxyfen to the body of adult mosquitoes. Nevertheless, the pupal mortality in the water from the Monitoring Ovitraps was significantly higher for the pyriproxyfen treatment than for the untreated control (Figure 5). Thus, it appears that horizontal transfer of pyriproxyfen by adult females in the Breeding Site Ovitraps contributes to pupal mortality.

In Experiment II, we observed a gradual decrease in pupal mortality over time (Figure 5). Takagi et al. [48] reported that pyriproxyfen remained effective against immature stages of *Ae. albopictus* for longer under laboratory conditions than under field conditions, because of differences in the amount of water contained in the ovitraps. Takagi et al. [48] added water to experimental containers, to avoid complete drying out during the study period. In the present study, the addition of water to the ovitraps may have diluted the pyriproxyfen. Moreover, decreased adult density in the microcosms may have contributed to a reduction in the transfer of pyriproxyfen into the larval breeding sites.

The results of the present study demonstrate that pyriproxyfen-treated bed nets offer significant potential to reduce *Ae. albopictus* populations, using the resting–migration behavior of adults in small microcosms (length, 270 cm; width, 270 cm; height 227 cm). Flight distances of *Ae. albopictus* are variant among populations. Niebylsky and Craig [49] reported that the maximum dispersal distance of *Ae. albopictus* at a scrap tire yard in Missouri, USA was 525 m. Marini [50] showed that the average of daily-mean distance traveled was 119 m and the maximum observed distance travelled ranged from 199 to 290 m in Rome, Italy. However, Takagi et al. [51] demonstrated that 90% of adult females were collected less than 36 m from the release point at the campus of Nagasaki University, Japan. Thus, strong efficacy of pyriproxyfen against the population used here would be expected within a range of ca. 30 m. Based on the flight distance of *Ae. albopictus* [49]–[52], the effectiveness of our present method might extend up to 500 m from the pyriproxyfen-treated source. Additionally, all blood-fed female necessarily entered into contact with pyriproxyfen, since all sources of food were confined inside the impregnated bed net in this study. However, in a field situation it is likely that a large portion of females would feed on blood outside the bed nets and, given the behavior of biting in hours of human intense activity, they could never touch on the bed nets. In this sense, the result of this study may be overestimated in open field experiment. Further field experiments are required quantitatively to investigate this.

*Aedes* mosquitoes use small water pools as breeding sites. The small amount of pyriproxyfen transferred by adult females could therefore result in concentrations high enough to cause the pupal mortality [14], [33], [36], [47]. In accordance with a previous laboratory study [32], [34], our present study confirms the effectiveness of pyriproxyfen against *Ae. albopictus* under semi-field conditions. There is concern that pyriproxyfen might affect the larval development of non-target organisms coexisting with the target mosquito species larvae [30]. However, the larvae of container-bred mosquitoes do not coexist with their predators [53]. Moreover, insect growth regulators generally have good safety margins for dominant macroinvertebrates and fish in mosquito breeding sites, and also low mammalian toxicity, because of the special mode of action of pyriproxyfen [54]–[57]. Thus, in the case of container-bred mosquitoes, the impact of this method of transfer on other organisms could be lower than an intensive application of conventional insecticide.

In conclusion, we have demonstrated that blood-fed adult female *Ae. albopictus* mosquitoes may be used as a vehicle to deliver pyriproxyfen into small and cryptic mosquito larval habitats. Furthermore, a reduction in fecundity and egg hatchability following contact with pyriproxyfen could effectively suppress the mosquito populations. The method described here constitutes a potential powerful tool for the future control of malaria, and also dengue, using bed nets or interior walls.
